# Programmable low-coherence wavefronts for enhanced localization

**DOI:** 10.1038/s44172-025-00502-6

**Published:** 2025-10-16

**Authors:** Burak Bilgin, Jy-Chin Liao, Hou-Tong Chen, Chun-Chieh Chang, Sadhvikas Addamane, Michael P. Lilly, Daniel M. Mittleman, Edward W. Knightly

**Affiliations:** 1 Department of Electrical and Computer Engineering, Rice University, Houston, TX USA; 2 Center for Integrated Nanotechnologies, Los Alamos National Laboratory, Los Alamos, NM USA; 3 Center for Integrated Nanotechnologies, Sandia National Laboratories, Albuquerque, NM USA; 4 School of Engineering, Brown University, Providence, RI USA

**Keywords:** Electrical and electronic engineering, Techniques and instrumentation

## Abstract

Engineering the properties of electromagnetic wavefronts has become essential to imaging, wireless security, sensing, and wireless communication. In particular, wavefronts that exhibit low spatial coherence can enable sensing functionalities with high accuracy and low latency. The typical use of such wavefronts cannot take advantage of these possibilities, as they require the ability to dynamically reconfigure the wavefront in a controllable and repeatable fashion, over a broad spectral bandwidth. Here, we propose a new approach for generating broadband reconfigurable wavefronts which not only exhibit low spatial coherence at a particular frequency, but are also decorrelated with the wavefronts simultaneously generated at other frequencies. We demonstrate that this frequency-domain decorrelation is a key feature that, in combination with dynamic reconfigurability, enables localization measurements with an order-of-magnitude improvement in accuracy compared to the state of the art.

## Abstract (web summary)

Burak Bilgin and colleagues propose an approach for generating broadband reconfigurable wavefronts exhibiting low spatial and spectral coherence. Broadcasting such wavefronts enables rapid localization with an order-of-magnitude improvement in accuracy compared to prior methods.

## Introduction

Wavefront engineering has become an integral method in the millimeter-wave and terahertz regimes for imaging, wireless sensing, security, and communications^1,2^. The ability to tailor and dynamically reconfigure the wavefront of an electromagnetic broadcast has emerged as a key capability for many RF systems. Starting with conventional beamforming using phased arrays, this field has recently grown to encompass more diverse and exotic wavefronts, including those which possess orbital angular momentum^3,4^ and those which exhibit unique and unusual properties in the near field of the transmitting aperture^5,6^. Of particular interest here is the class of wavefronts with low spatial coherence, in which the amplitude and/or phase of the wave *E*(*θ*, *ϕ*) vary rapidly with the angular coordinates. Such waves, initially described in the context of statistical optics^7^, have been recognized as valuable tools in RF systems, enabling advances in wireless security^8–11^, imaging^12–14^, and sensing^15–19^. Despite the growing interest in such wavefronts, there have been few examples exploiting the ability to dynamically reconfigure between different realizations of a low-coherence wavefront. This is particularly the case for broadband signals where diverse incoherent wavefronts are generated simultaneously across a bandwidth, allowing one to exploit not only the low spatial coherence but also the low coherence in the spectral domain.

In this work, we demonstrate that such dynamically reconfigurable low-coherence broadband wavefronts can provide unique new capabilities. We illustrate this point by demonstrating how such fields can be employed for enhanced angular localization, an important capability for many RF systems^20–23^. A crucial aspect of our approach is that each broadband wavefront is not only *spatially* incoherent but also *spectrally* incoherent, such that different frequency components within the bandwidth of the radiated signal exhibit distinct angular amplitude and phase profiles. The aperture that generates these wavefronts is equipped with resonators that have continuously tunable and frequency-diverse amplitude and phase modulation capabilities, enabling sequential re-randomization of the wavefronts at each frequency, thereby unlocking distinct low-coherence wavefront generation across different time instances over wideband. In this work, we exploit this joint spectral and spatial coherence to demonstrate a new framework for angular localization, which offers better than 0.1° angular uncertainty, representing an order-of-magnitude improvement in estimation error with respect to prior methods^15,24–26^, with the lowest average error reported being 1° in ref. ^26^.

## Results and discussion

### Wavefront generation

**Fig. 1:**
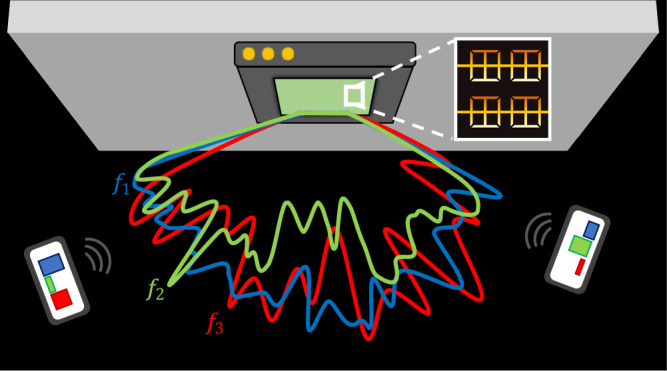
Low-coherence wavefronts. Visualization of a broadband source generating low-coherence wavefronts at different frequencies simultaneously by electrically tuning the metasurface attached to its transmitting aperture. Its tunability allows dynamic wavefront generation and deployment of effective communication tools through the same structure, enabling joint communication and sensing.

Our approach for generating programmable low-coherence wavefronts combines a wideband radiation source with a multi-pixel transmissive metasurface, as illustrated in Fig. 1. The metasurface consists of an array of sub-wavelength metallic split-ring resonators (SRRs) on a dielectric substrate. These SRRs are grouped into interconnected subsets to form a number of adjacent column-shaped pixels^27^. Each electrically-interconnected set of SRRs forms a Schottky contact to the underlying doped semiconductor, such that a reverse bias applied between this contact and an ohmic contact generates a depletion region underneath and adjacent to the metallization. An analog voltage applied to the Schottky contact, therefore, continuously tunes the scattering response of the column of SRRs to an incident THz wave^28^.

The geometrical parameters of the SRRs are chosen to produce a resonant response at a frequency of 145 GHz, producing an amplitude modulation over a spectral width of about 25 GHz, and an on-off contrast ratio of about 4.5 dB on resonance. As a result, in the waveguide D band (frequencies between 110 and 170 GHz), both the amplitude and phase of the field scattered by the SRRs change with respect to both frequency and applied bias. The pixels, therefore, collectively form a one-dimensional reconfigurable grating, where the scattering response of each column of the grating can be independently controlled by an external voltage bias^27^. For any voltage *V* applied to a given column within the allowable voltage range (between zero volts and  −20 V, the maximum reverse bias), the scattered field from that column experiences an amplitude change Δ*A* and a phase shift Δ*ϕ* which both depend on the voltage, and which vary for each frequency within the incident wave’s spectral bandwidth (see Supplementary Note 1 and Fig. S1). By applying a random set of control voltages ${{{{\mathcal{V}}}}}=\{{V}_{1},\,{V}_{2},\,...,\,{V}_{P}\}$ to the *P* columns, we can therefore convert an incident spatially coherent beam into an outgoing scattered wave with low spatial coherence in one dimension (i.e., in the plane perpendicular to the columns of the metasurface).

Evidently, the efficacy of this approach to generating low-coherence wavefronts depends on the range of amplitude and phase values at each of the incident frequencies, Δ*A*_*max*_(*f*) and Δ*ϕ*_*max*_(*f*). One challenge in implementing this strategy for producing low-coherence wavefronts lies in the achievable range of modulation parameters provided by the metasurface. The limited range of phase modulation is a well-known problem in such planar devices^29^, with no examples in the literature reporting greater than about Δ*ϕ*_*max*_≈130°^30^ (although more elaborate designs can achieve greater than 2*π* range^31^). For our device, the maximum phase modulation depends on frequency due to the resonant response, but never exceeds about Δ*ϕ*_*max*_≈50° in the D band spectrum. We therefore must determine the minimum spatial coherence that can be produced by a modulator of the sort described here.

### Numerical modeling

As a first step, we explore this question using Monte Carlo simulations. For a given range of amplitude and phase modulation Δ*A*_*max*_ and Δ*ϕ*_*max*_, and a given number of pixels *P*, we model each pixel (column) of the device as a scattering site producing a spherical wave with a randomly chosen amplitude and phase within the allowable range. We then compute the superposition of these spherical waves to determine the net electric field as a function of angle, according to:

1 $$E(f,\theta )=\sum\limits_{p=1}^{P}\frac{1}{{r}_{p}}{e}^{i\omega {r}_{p}/c}\,{A}_{p}{e}^{i{\phi }_{p}}\,{A}_{in}{e}^{i\omega t}$$

Here, *r* = {*r*_1_, *r*_2_, ⋯  *r*_*P*_} is the vector of distances from the *pth* column to the user, *A*_*in*_*e*^*iωt*^ is the incident wave (a uniform plane wave), and ${A}_{p}{e}^{i{\phi }_{p}}$ denotes the scattering function of the *pth* pixel, chosen randomly within the allowed range defined by Δ*A*_*max*_ and Δ*ϕ*_*max*_. Since the metasurface in our study contains long, narrow column-shaped pixels, we may treat this as effectively a one-dimensional problem, neglecting the azimuthal angle dependence, although of course the results can readily be generalized to two-dimensional metasurface arrays.

For each *E*(*f*, *θ*) determined in this fashion, we can compute the degree of angular correlation of the wavefront. By averaging over many realizations of the randomness, we can extract an ensemble-averaged angular correlation function, defined as:

2 $$C(\Delta \theta )=\frac{\langle E({\theta }_{1}){E}^{* }({\theta }_{2})\rangle }{\sqrt{\langle | E({\theta }_{1}){| }^{2}\rangle \langle | E({\theta }_{2}){| }^{2}\rangle }}$$

where the angle brackets indicate averaging over the random ensemble, and where Δ*θ* = *θ*_1_ − *θ*_2_ (see “Methods” section for details on ensemble averaging).

**Fig. 2:**
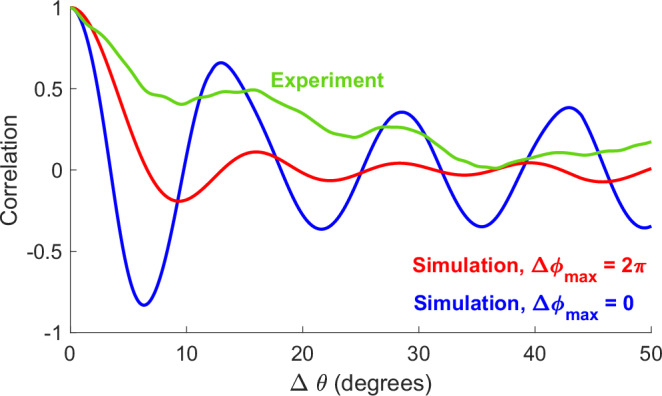
Angular correlation characterization. Average correlation function at calculated according to Equation (2) at 145 GHz and varying angular separation values for an ideal phase-only metasurface with a full range of phase modulation (Δ*ϕ*_*max*_ = 2*π*, red), no phase modulation (Δ*ϕ*_*max*_ = 0, blue), and measured using the real metasurface (green). In the simulations, the pixels of the metasurface are assigned randomized phase responses within the allowed range: [0, Δ*ϕ*_*max*_], and then the complex E-field *E*(*θ*) is calculated. For Δ*ϕ*_*max*_ = 2*π*, the correlation function is averaged over 1000 randomly chosen configurations. The experimental data is measured and averaged over 10 configurations.

Figure 2 depicts typical results of such simulations with an ideal phase-only metasurface, showing the variation of *C*(Δ*θ*) for two extreme cases: when the phase modulation range Δ*ϕ*_*max*_ is zero (i.e., no variation at all across the metasurface) and 2*π* (the full range of possible values for each pixel). From these results, we observe that the average angular correlation oscillates when the metasurface’s modulation range is limited, showing large correlation even at large values of Δ*θ*. On the other hand, when the metasurface has full 2*π* phase control, the average correlation quickly decays to zero and stays near zero with increasing Δ*θ*, showing that the dynamic range of the metasurface plays a critical role in generating wavefronts with low angular coherence.

Having established the average correlation behavior in such extreme cases, we then evaluate the low-coherence wavefront generation capability of the metasurface used in our experiments, by applying the same correlation function to experimentally measured wavefronts (green curve in Fig. 2). These experimental results show weaker oscillatory features than the Δ*ϕ*_*max*_ = 0 simulation, as well as a slower correlation decay compared to the Δ*ϕ*_*max*_ = 2*π* simulation. In other words, the experimental result exhibits behavior that is intermediate between the two simulated cases.

These results call attention to the consequences of the limited phase modulation range of most metasurface devices, noted above. Although an ideal metasurface can produce a very low-coherence wavefront, the real devices achieve a slower decay to zero correlation. This slower decay ultimately limits the ability of such devices to achieve high-accuracy localization^15,16^. To overcome this limitation, we consider the possibility that our incident wave may be spectrally broadband, so that we can exploit not only low spatial coherence at a single frequency but also low correlation between different frequency components.

### Broadband characterization

**Fig. 3:**
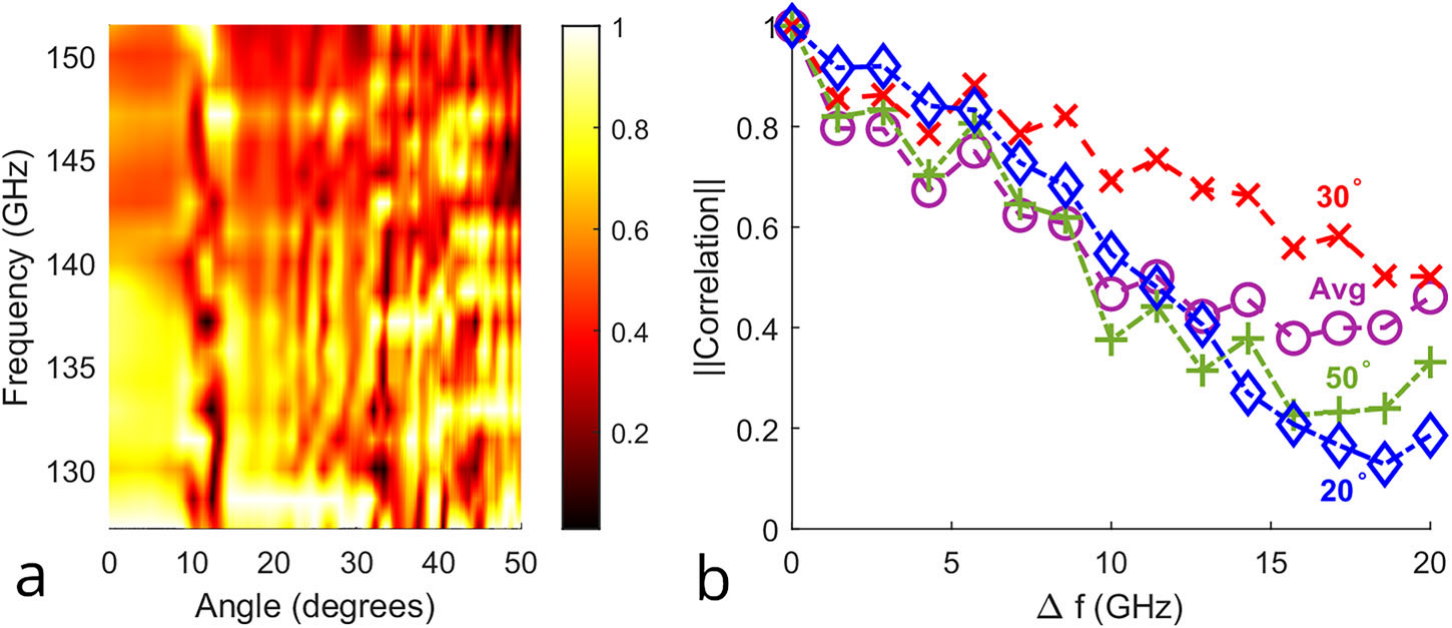
Wideband wavefront characterization. **a** Normalized linear amplitude values at varying angles over a 24.3 GHz bandwidth generated by a metasurface configured to generate low-coherence wavefronts as characterized in Equation (1), with a particular fixed (but randomly chosen) set of control voltages applied to the pixels of the device. To emphasize the frequency decorrelation at each angle, the broadband signal obtained at each angle is normalized independently. The frequency-diverse response of the metasurface is evident in the random variation of the heatmap along the vertical direction. **b** The absolute value of correlation calculated at varying frequency separation values, with a frequency step size of 1.43 GHz (determined by the spectral resolution of the measurement apparatus). Three representative curves are shown for specific angular locations (blue at 20°, red at 30°, green at 50°), while the purple curve shows the average.

As noted previously, the scattering function *Ae*^*iϕ*^ of the pixels is frequency-dependent. Consequently, a single bias voltage corresponds to a diverse set of scattering functions. This frequency-diverse behavior of the metasurface is a critical enabler of generating low-coherence wavefronts simultaneously across various frequencies when a broadband source illuminates the surface. Fig. 3a presents an example, generated by illuminating the metasurface with a broadband input, with a single fixed (randomly chosen) set of voltages applied to the pixels. The vertical striations in this heatmap suggest some correlated behavior across frequency. So, as above in Fig. 2, we can quantify the correlation decay. Here, instead of calculating correlation over a set of angular separation values of the same wavefront, we calculate it across different wavefronts generated simultaneously at different frequencies, separated by Δ*f*, at a fixed angular location. We then average this behavior over multiple random voltage configurations. Fig. 3b shows the results for several fixed angular locations, as well as the average. These results show that the frequency-diverse response of the metasurface, combined with a broadband source signal, can generate a set of wavefronts that exhibit decorrelation across both spectral and spatial dimensions.

We note that while the results in Fig. 3b demonstrate the average behavior of multiple metasurface configurations, it would also be of interest to study the diversity of the behavior across different configurations. On the other hand, due to the large number of possible configurations (~10^37^) and the requirement of identifying the subset of these configurations that are valuable for the purposes of this work, we defer this exploration to future work.

#### Angular localization

In the preceding sections, we demonstrated a method to generate wavefronts that exhibit decorrelation in both spatial and spectral domains. Next, we consider the use of these dynamically reconfigurable low-coherence broadband wavefronts for sensing. We envision that a wireless communication node could use these wavefronts as beacons to enable the angular localization of a target receiver. Specifically, spatial decorrelation enables the unique characterization of different angles within the field of view, while spectral decorrelation enables the expansion of this characterization to many distinct frequencies, thus creating a wide “feature” space. If we discretize the angles within the transmitter’s field of view to *K* different values, Θ = {*θ*_1_, *θ*_2_, . . . , *θ*_*K*_}, then each of the *F* frequency components within the transmitted bandwidth {*f*_1_, *f*_2_, . . . *f*_*F*_} corresponds to an element *h*(*f*, *θ*) of a one-shot signature codebook *H*_*KF*_, which can be pre-characterized, and used by the receiver to perform an angle-of-departure (AoD) estimation.

A user located at an angle of departure *ϕ* receiving the broadband signal $\overline{h}=[\overline{h}({f}_{1},\phi ),\ \overline{h}({f}_{2},\phi ),\ ...,\ \overline{h}({f}_{F},\phi )]$ can obtain an estimate of the angle of departure, ϕ^*^, by identifying the closest pre-characterized codebook signature *h*(*θ*) using a simple minimization procedure:

3 $$\phi^{*} = \arg {\min} _{\theta \in {{\Theta}}} \left\|{\bar{h}}-h(\theta)\right\|_2^2$$

We emphasize that this is a low-latency measurement since all of the signatures *h*(*f*_*j*_, *ϕ*) are transmitted simultaneously.

Furthermore, in scenarios with low signal-to-noise ratio (SNR) or bandwidth limitations, we can perform repeated measurements to improve the accuracy of the localization result. By changing the *P* voltages applied to the metasurface columns from one random set to a different random set, we reconfigure the metasurface to produce a different set of wavefronts, also exhibiting low coherence but distinct from the first set. We can repeat this procedure *T* times, using voltage configurations $\{{{{{{\mathcal{V}}}}}}_{1},\ {{{{{\mathcal{V}}}}}}_{2},\ ...,\ {{{{{\mathcal{V}}}}}}_{T}\}$. Thus, by generating new sets of signatures *h*_*t*_(*f*_*j*_, *ϕ*), (*t* = 1, 2, . . . , *T*) sequentially, we expand our codebook from frequency-domain only (*H*_*KF*_) to both frequency and time (*H*_*KFT*_), thus enabling the scaling of estimation accuracy in both domains.

Given the limited ability of metasurfaces to produce low-coherence wavefronts due to their limited modulation range, as characterized above, the efficacy of this localization procedure must be demonstrated. The spatial correlation decays (see Fig. 2) suggest an average speckle spot size of a few degrees, so it is not obvious that one could achieve sub-degree precision in angular localization. Yet, the ability to exploit broadband signals offers an opportunity that warrants further study.

To evaluate the performance, we conduct experiments using a time-domain THz spectroscopy system^32^. A broadband beam with a nearly ideal Gaussian spatial profile illuminates the metasurface at normal incidence, and a receiver detects the scattered low-coherence wavefront, as a function of angle, in a transmission geometry. Due to the low power of time-domain THz systems, these measurements are carried out at a small scale over a relatively short range; the general approach, however, is scalable with higher power to arbitrary transmitter–receiver distances. We can estimate the angle of departure using these measured signals, discretized with 0.1° resolution, via Equation (3). We perform one set of measurements to build the codebook using 10 randomly chosen voltage sets, and then repeat the measurements using the same voltage sets as a test measurement. We compute the error between the ground-truth angular position of the receiver and the estimated value, using different numbers of spectral components (codebook bandwidth) and numbers of measurements (codebook time steps).

**Fig. 4:**
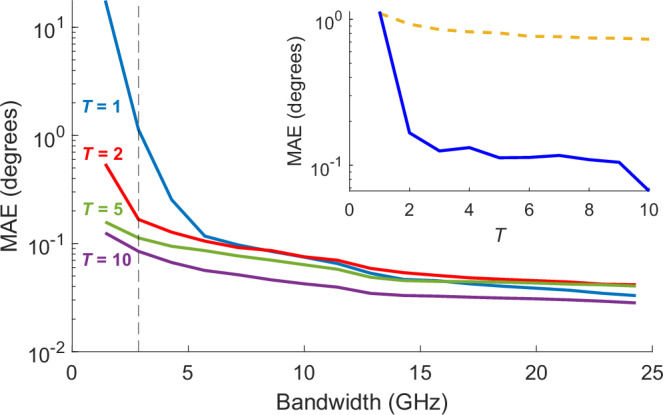
Angle estimation with varying time and frequency resources. The mean absolute error (MAE) achieved with varying bandwidth values used to construct the angle estimation codebook. By varying the codebook variable *T*, we perform angle estimation ranging from one-shot to 10-shot (see “Methods” section). In the figure, the blue, red, green, and purple curves represent *T* = 1, 2, 5, and 10, respectively. The MAE is calculated by averaging the estimation error for all beacons that correspond to the entire FoV and then averaging over 100 independent noise observations at a signal-to-noise ratio (SNR) of 30 dB. As the collected signals are discretized with 0.1^∘^ resolution, the error for estimation is also calculated with 0.1^∘^ discretization, e.g., estimations of signals assigned in the correct angle “bin" correspond to zero error. The inset shows the dramatic improvement in estimation error resulting from repeated measurements (increasing *T*) with a solid blue curve. Also shown (dashed yellow curve) is the improvement that would result if the only effect of repeated measurements was to improve the overall SNR through averaging.

Figure 4 shows the mean absolute error (MAE) as a function of codebook bandwidth, where each curve corresponds to a different number of iterated measurements (*T*). Even for a single measurement (*T* = 1), and despite the correlated behavior across angular separations of a few degrees (Fig. 2) and across spectral separation of a few GHz (Fig. 3b), we nevertheless observe that the error decreases rapidly with increasing number of spectral components. For instance, the MAE decreases from 17.7° with only two complex wavefront components in the codebook to 0.033° with 18 components across the spectral range 127–152 GHz. We note that an MAE of less than 0.1° represents more than one order-of-magnitude improvement compared to the state of the art in AoD estimation^15,24–26^.

Furthermore, as we enable sequential randomization of the wavefronts by reconfiguring the metasurface, we observe (inset of Fig. 4) that the estimation error is also improved with increasing *T*. This improvement is more pronounced at lower bandwidth values (<5 GHz) since there are only a few (<5) frequency components in the beacon. While still notable, the improvement is less pronounced at higher bandwidth, where the estimation accuracy with 10 shots reaches saturation around 0.03° with 24.3 GHz bandwidth. However, it should be noted that the saturation is dependent on other factors such as the signal-to-noise ratio (SNR), as discussed below. Improving estimation error through increasing bandwidth and number of shots provides the users with a time-frequency trade-off, thereby allowing accurate angle estimation even in bandwidth-contentious networks.

### Noise robustness

**Fig. 5:**
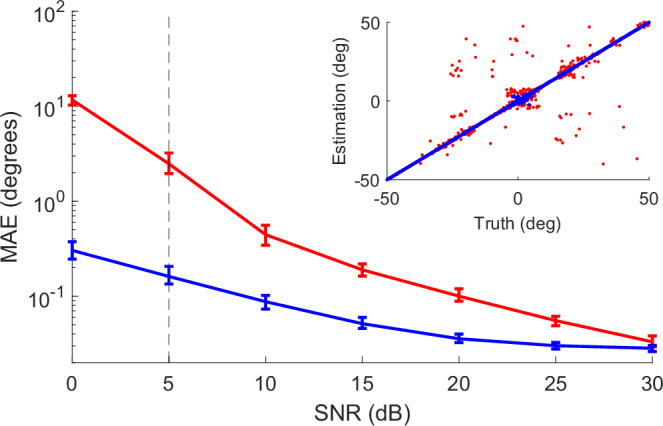
Noise robustness of angle estimation. Mean absolute error (MAE) values (on a log scale), calculated over the entire field of view using the full 24.3 GHz codebook bandwidth, at varying signal-to-noise ratio (SNR) levels. Red and blue curves correspond to one-shot and 10-shot (*T* = 1 and 10), respectively. Error bars represent the minimum and maximum errors within 100 independent estimations. The inset shows two example sets of estimated angle values against ground truth at 5 dB SNR for one-shot and 10-shot estimation.

The above analysis assumes a particular level of noise (SNR = 30 dB), and our results must clearly depend on this value since the received signal $\overline{h}$ is a noisy and attenuated version of the pre-characterized beacon *h*(*θ*). To evaluate the robustness of our approach against noise, we compare the MAE values at varying SNR levels for one-shot (*T* = 1) and 10-shot (*T* = 10) estimation (Fig. 5). Here, degradation in estimation accuracy with decreasing SNR occurs much faster for one-shot estimation compared to 10-shot. Specifically, for an SNR of 5 dB, one-shot estimation results in 2.5° MAE, an order-of-magnitude higher than that of 10-shot estimation. On the other hand, at 30 dB SNR, the respective MAE values are quite similar (see “Methods” section for details on added noise).

To understand how a low SNR affects individual angle estimations, we show in the inset of Fig. 5 two example sets of estimations against the ground truth for one-shot and 10-shot estimation methods, for an SNR of 5 dB. Here, a perfectly accurate estimation profile aligns with *y* = *x*, and our estimation profiles at high SNR = 30 dB for one-shot and multi-shot methods closely follow this trend (the 30 dB SNR results are shown in Supplementary Note 2 and Fig. S3). However, at a much lower 5 dB SNR (inset), clear differences are observable. The estimated values with 10 shots (blue points) are still close to the ground truth, scattered near *y* = *x*. In contrast, the single-shot estimations (red points) exhibit some scatter throughout the field of view. In particular, two distinct patterns can be identified: many of the estimated points are clustered near the *y* = *x* ground truth, while those that are not appear to be scattered roughly symmetrically about the opposite line *y* = −*x*. This pattern reflects an underlying mirror symmetry in the generated wavefronts. This symmetry has one obvious source: the edges of the metasurface. As noted above, the metasurface consists of a series of adjacent parallel columns, which collectively define a rectangular aperture through which the normally incident Gaussian beam is diffracted. This aperture diffraction pattern, symmetric around *θ* = 0°, is superimposed on the spatially random fluctuations induced by the randomly configured metasurface. This source of left-right correlation could be eliminated through careful design of both the metasurface and its packaging, as well as more careful apodization of the input beam. Yet, despite this effect, we emphasize that our method can still achieve a remarkable MAE of only 0.16° with a 10-shot estimation protocol, even with a low SNR of 5 dB.

## Conclusions

We have described a novel method to dynamically generate broadband wavefronts with low coherence in both angle and frequency, and demonstrated that this capability can enable low-latency estimation of angle of departure, with unprecedented accuracy. A key aspect of this innovation lies in the repeatable reconfigurability of the metasurface. Unlike a wireless channel in a rich multi-path scattering environment, here the low-coherence wavefront can be repeatably generated. This enables the idea of using a pre-characterized codebook for localization. This, of course, relies on the fact that the channel in our envisioned use case is dominantly line-of-sight, with little contribution from multi-path effects, which is often the case for frequencies above 100 GHz^20^.

The low-coherence wavefronts employed in our work should be contrasted with the smooth convex angle-frequency function of a THz leaky-wave antenna (LWA), which has been used in prior work^24–26,33,34^ to demonstrate a low-latency (one-shot) localization, also in a line-of-sight channel, by exploiting the dispersive angle-frequency coupling. That approach results in limited accuracy due to the strongly correlated response at adjacent angles, and cannot be significantly improved by using sequential measurements. Similarly, other static structures^15,16,35^ discussed previously for one-shot localization are not capable of carrying out dynamic wavefront generation. In contrast, the reconfigurability of our metasurface allows us to produce a sequence of distinct low-coherence wavefronts, leading to dramatic improvements with iterated measurement. Thus, our approach offers a unique ability to engineer the trade-off between the latency (number of shots) and localization accuracy, and achieves extremely high accuracy even for a relatively small number of iterations (≤10) and even in relatively poor SNR environments. Indeed, this method can achieve a mean absolute error better than 1° even in a channel with an SNR of 0 dB, and better than 0.1° at higher SNR in a single shot. Due to the fact that we exploit the rapid correlation decay in both space and frequency, we not only achieve a finer-resolution system, but also demonstrate these remarkably precise values for localization, even though the angular correlation function (for a given frequency) decays much more slowly.

We also note that LWAs have been proposed in prior work as promising candidates for communication networks^25,36–38^. However, the communication functionality of this architecture is severely limited by its dispersive and non-reconfigurable radiation pattern. In contrast, the reconfigurable architecture in our work unlocks critical integrated communication and sensing capabilities that were previously not available through LWAs.

Finally, we emphasize that our experimental demonstrations employ a metasurface with a relatively limited phase modulation range of only Δ*ϕ*_*max*_ ≈ 50°. The achieved accuracy and the rate of convergence with increasing bandwidth both depend on the characteristics of the metasurface scattering function ${A}_{p}{e}^{i{\phi }_{p}}$, including both the range of phase modulation and its frequency dependence. The square SRR configuration employed in our system does not offer the largest demonstrated range of values for these parameters^30^, so further improvements are clearly possible. Furthermore, since the wavefronts generated in our system are broadcast, the same principle would work in the presence of multiple receivers: each receiver, presumably at a different location from others, would be able to identify its corresponding angle of departure without interference effects. Similarly, as the constructed codebooks would be affected by changes to metasurface-to-receiver distance, we believe that this could be further exploited for simultaneous angle and distance estimation with a further optimized pool of metasurface configurations and the receiver’s estimation algorithm. Consequently, studying the characteristics of the generated wavefronts at varying propagation distances will be an integral part of expanding this work. In addition, studying the wavefronts in azimuth and elevation jointly will be critical to accurately model the three-dimensional field of view, thus enabling full positioning of the receiver. As such, we believe these are interesting avenues to explore in future work.

Nevertheless, it is remarkable that such precise results can be obtained even with significant limitations on the modulation parameters.

## Methods

### Experimental setup

We use a LUNA T-Ray 5000 Time-Domain Terahertz Spectroscopy system with a single transmitter and receiver producing directional, approximately Gaussian beams^32^. In the setup (shown in Supplementary Fig. S2), the metasurface is placed 11 centimeters away from the stationary transmitter and is centered on top of a motorized stage to enable fine-resolution angular positioning. The receiver is placed 15 centimeters away from the metasurface on a rail attached to the motorized stage. Due to the ultra-low power architecture of the system (~10 μW spread across  ~2 THz bandwidth^25^), the limited modulation efficiency of the metasurface, and the fact that the source power is scattered over a wide angle range, the distances are relatively small, to avoid the effect of experimental noise on the generated random spectral patterns.

To generate the wavefronts, we obtain an amplitude-voltage curve at the resonance frequency, 145 GHz, according to the experimentally observed amplitude response-bias voltage relation in Supplementary Fig. S1b. Then, we select 16 amplitude response values randomly, with a uniform distribution, from the range of possible amplitude response values. Using the previously obtained amplitude-voltage curve, we then convert these 16 amplitude values to their corresponding voltage values, which make up a single voltage configuration. By applying this voltage configuration to the metasurface’s pixels, we generate the described low-coherence wavefronts upon excitation from the wideband THz source. We select 10 different configurations to evaluate the localization performance at up to *T* = 10 shots.

To collect the data, we first apply the designed set of voltages to the functioning pixels (columns) of the metasurface using a National Instruments DAC card (NI PCIe-6738). Then, we rotate the receiver across the metasurface’s field of view Θ = [−50°, 50°] with 0. 1° resolution and collect the received time-domain signal at each location, with the signal averaged 100 times to ensure high experimental SNR. Time-domain waveforms are Fourier transformed to extract spectral information (amplitude and phase of the measured electric field) for all frequencies within the bandwidth of the measured signals. For the measurements described here, we focus only on a subset of this broad spectrum, corresponding to the range 127–152 GHz, the range over which the metasurface provides an amplitude modulation tunable with applied DC bias (see Supplementary Fig. S1b). For each set of voltages, the same dataset is collected twice this way across the entire field of view, once for codebook construction and the other as a test set.

### Signal processing

For the calculation of the correlation function in Fig. 2, near-zero angles are excluded from the ensemble averaging to avoid the effect of unmodulated signal leakage. For angle estimation (Figs. 4 and 5), we add white Gaussian noise to all frequency components of each received signal in the test set. The SNR is defined with respect to the average power from all frequency components of a given signal. For the analysis in Fig. 4, the added noise is adjusted with SNR = 30 dB. In the inset, the SNR is 30 dB for the curve depicting the improvement from adding shots (blue), while it ranges between 30 dB and 35 dB for the curve depicting the improvement from simple averaging (yellow dashed), reflecting the SNR change resulting from such averaging. For Fig. 5, it is varied between 0 and 30 dB, and the estimation profiles in the inset are achieved at 5 dB. As an estimation algorithm, we use the Least Squares Orthogonal Matching Pursuit (LS-OMP)^39^ algorithm with known sparsity (1-sparse), which is equivalent to the least squares error comparison between the received signal and each column of the codebook. We estimate the angle of each received signal in the test set and calculate the mean absolute error (MAE) across the field of view, then repeat 100 times with different noise observations to calculate the mean and variance of MAE.

## Supplementary information

Supplementary Information

## Data Availability

All relevant data are available from the corresponding author upon reasonable request.
