# Supplementary material for: Programmable low-coherence wavefronts for enhanced localization
Source: Commun Eng. 2025 Oct 16;4:179. doi: 10.1038/s44172-025-00502-6 (PMC12533259; doi:10.1038/s44172-025-00502-6)
Supplement: Supplementary file 1 — Supplementary Information [file 44172_2025_502_MOESM1_ESM.pdf]

Supplementary Materials

Programmable low-coherence wavefronts for enhanced localization

Burak Bilgin[1], Jy-Chin Liao[1], Hou-Tong Chen[2], Chun-Chieh Chang[2], Sadhvikas Addamane[3], Michael P. Lilly[3], Daniel M. Mittleman[4], Edward W. Knightly[1]

[1]Department of Electrical and Computer Engineering, Rice University, Houston, TX, USA.
[2]Center for Integrated Nanotechnologies, Los Alamos National Laboratory, Los Alamos, NM, USA.
[3]Center for Integrated Nanotechnologies, Sandia National Laboratories, Albuquerque, NM, USA.
[4]School of Engineering, Brown University, Providence, RI, USA.

Supplementary Note 1. Metasurface Architecture

We use a planar metasurface to generate low-coherence wideband wavefronts. The 2.5 cm x 2 cm metasurface (shown in Figure S1a) is fabricated by standard photolithography on a 2 μm-thick n-doped GaAs layer (0.5×10^{16} carriers per cm^3) grown on a semi-insulating GaAs substrate [1]. The electrically-connected gold split-ring resonators and the n-doped GaAs layer, along with the adjacent ohmic contacts yield a Schottky junction structure, in which the carrier density of the n-GaAs at the split gaps of each resonator can be tuned by reverse biasing the resonators, enabling continuous and dynamic tuning of the amplitude and phase response of the resonator to incident sub-THz EM waves [2, 3]. The metasurface is divided to 16 column-pixels and each pixel consists of 6 x 155 electrically-connected resonators. Different pixels are electrically disconnected so that independent voltage biases can be applied to tune their resonant response. The resonators are designed to have an outer dimension of 163 μm, metal line width of 6 μm, and gap width of 2 μm, which gives rise to the lowest-order LC resonance at 145 GHz. We note that the metasurface has an insertion loss of 9.4 dB [3], and is not optimized in this respect. For example, part

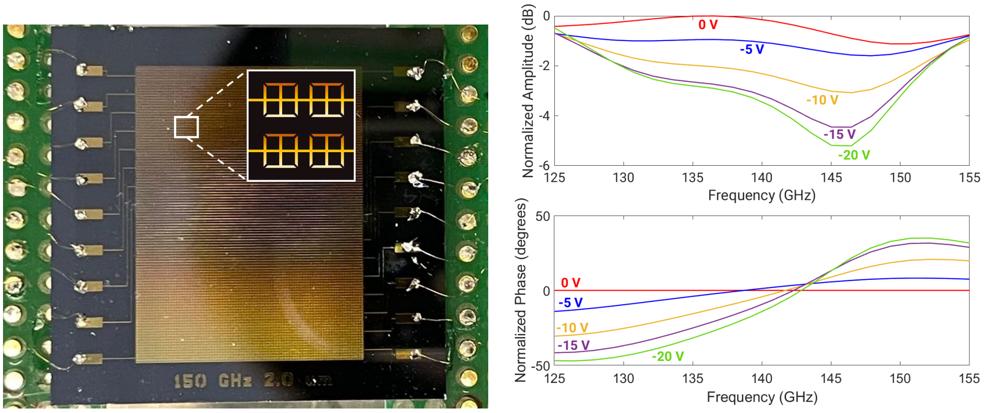

(a) (b)

Supplementary Fig. S1: **(a)** Fabricated metasurface. The amplitude and phase response of each of the 16 pixels is independently controlled through the shown voltage channels. The applied voltage determines the free carrier density in the depletion region of the resonators (inset) in each pixel, allowing continuous tuning of the resonance characteristics. **(b)** The broadside amplitude and phase response range of the metasurface when a uniform voltage is applied to all of the pixels. While only the amplitude and phase response values for 5 voltage values are shown (-20, -15, -10, -5, and 0V with green, purple, yellow, blue, and red curves respectively), the response is continuously adjustable through adjusting the voltage. Both amplitude and phase responses in this band are frequency-diverse, enabling wavefronts in generated in nearby frequencies to be minimally correlated.

of the insertion loss is a result of back-surface reflection, and an anti-reflection coating could be added to reduce the loss considerably. The transmission amplitude and phase spectra of the metasurface exhibit complementary tuning responses when all 16 pixels are tuned with a uniform bias voltage, shown in Figure S1b within the frequency range of interest in this work, thus enabling frequency-diverse amplitude and phase modulation over the 127-152 GHz band.

We note that the rate at which the metasurface can be reconfigured from one configuration to another is on the scale of several kHz [4]. In the case of continuous location estimation, i.e., target tracking, this switching rate would be the limiting factor in multi-shot acquisition. We believe that the millisecond switching speed of our metasurface is sufficient for most real-life moving targets, at least in indoor environments. We also note that faster metasurfaces have been demonstrated, so this number is by no means a fundamental limit. A similar architecture with 100 MHz switching speed was shown in [5].

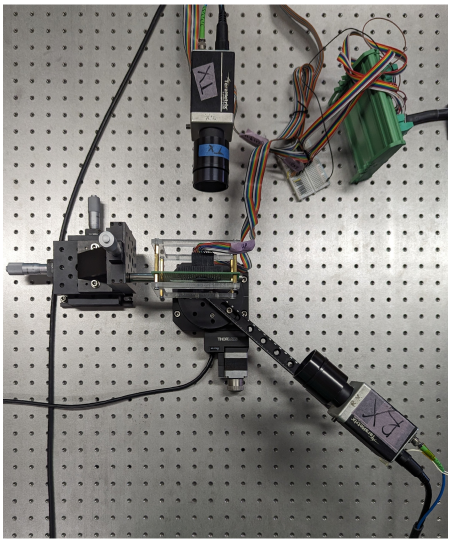

Supplementary Fig. S2: Full experiment setup with LUNA T-Ray 5000 Time-Domain Terahertz Spectroscopy system, and the metasurface.

Supplementary Note 2. Angle Estimation Profiles at 30 dB SNR

A example set of estimated angle values against ground truth at 30 dB SNR for one-shot and 10-shot estimation are shown in Figure S3.

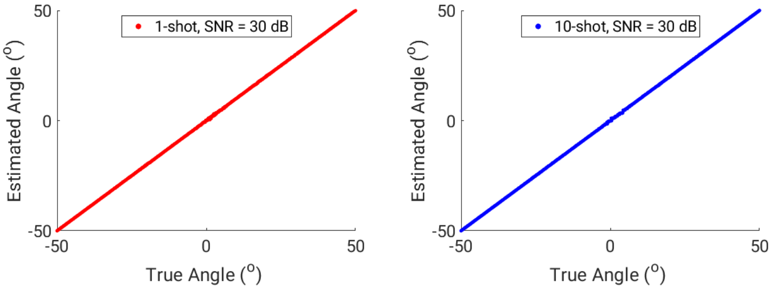

Supplementary Fig. S3: Similar to the estimation profiles shown in the inset of Figure 5 in the main text, we show here two example estimation profiles for 1-shot and 10-shot schemes at 30 dB SNR. Here, it is clear that both schemes achieve low-error angle estimation, thus exhibiting a y=x trend.

References

[1] Chen, H.-T. *et al.* Active terahertz metamaterial devices. *Nature* **444**, 597–600 (2006).

[2] Chen, H.-T. *et al.* A metamaterial solid-state terahertz phase modulator. *Nature Photonics* **3**, 148–151 (2009).

[3] Karl, N. *et al.* An electrically driven terahertz metamaterial diffractive modulator with more than 20 dB of dynamic range. *Applied Physics Letters* **104**, 091115 (2014).

[4] Shaikhanov, Z. *et al.* Audio misinformation encoding via an on-phone subterahertz metasurface. *Optica* **11**, 1113–1114 (2024).

[5] Lan, F. *et al.* Real-time programmable metasurface for terahertz multifunctional wave front engineering. *Light: Science & Applications* **12** (2023).
